# Cell death in head and neck cancer pathogenesis and treatment

**DOI:** 10.1038/s41419-021-03474-5

**Published:** 2021-02-18

**Authors:** Martina Raudenská, Jan Balvan, Michal Masařík

**Affiliations:** 1 Department of Physiology, Faculty of Medicine, Masaryk University / Kamenice 5, CZ-625 00 Brno, Czech Republic; 2 Department of Chemistry and Biochemistry, Mendel University in Brno, Zemedelska 1, CZ-613 00 Brno, Czech Republic; 3 Department of Pathological Physiology, Faculty of Medicine, Masaryk University / Kamenice 5, CZ-625 00 Brno, Czech Republic; 4 BIOCEV, First Faculty of Medicine, Charles University, Prumyslova 595, CZ-252 50, Vestec, Czech Republic

**Keywords:** Oral cancer, Cell death, Oncogenesis

## Abstract

Many cancer therapies aim to trigger apoptosis in cancer cells. Nevertheless, the presence of oncogenic alterations in these cells and distorted composition of tumour microenvironment largely limit the clinical efficacy of this type of therapy. Luckily, scientific consensus describes about 10 different cell death subroutines with different regulatory pathways and cancer cells are probably not able to avoid all of cell death types at once. Therefore, a focused and individualised therapy is needed to address the specific advantages and disadvantages of individual tumours. Although much is known about apoptosis, therapeutic opportunities of other cell death pathways are often neglected. Molecular heterogeneity of head and neck squamous cell carcinomas (HNSCC) causing unpredictability of the clinical response represents a grave challenge for oncologists and seems to be a critical component of treatment response. The large proportion of this clinical heterogeneity probably lies in alterations of cell death pathways. How exactly cells die is very important because the predominant type of cell death can have multiple impacts on the therapeutic response as cell death itself acts as a second messenger. In this review, we discuss the different types of programmed cell death (PCD), their connection with HNSCC pathogenesis and possible therapeutic windows that result from specific sensitivity to some form of PCD in some clinically relevant subgroups of HNSCC.

## FACTS

- How cancer cells die is very important because the specific type of cell death has different immunomodulatory impact crucial for the therapeutic response.
- Scientific consensus describes many different cell death subroutines with different regulatory pathways and cancer cells are not able to avoid all of cell death types at once.
- Resistance to one type of cell death can confer sensitivity to another type.
- Specific sensitivity of cancer cells to some form of programmed cell death provides an interesting therapeutic window.
- Human papillomavirus (HPV) can interfere with some cell death regulatory pathways.

## OPEN QUESTIONS

- How do different cell death regulatory pathways interact with each other and with secretory and/or endocytic pathways?
- Is a certain genetic background in cancer cells tightly related to specific resistance and sensitivity to different kinds of cell death?
- Can be different types of programmed cell death modulated and switched?

## Introduction

Head and neck squamous cell carcinomas (HNSCC) are the fifth most common cancer globally. They emerge in the upper aero-digestive tract (including the oral cavity, pharynx and larynx). HNSCC exhibit high levels of heterogeneity and deep differences in therapeutic response^1^. Conventional HNSCC classification and clinical management are mainly based on clinical staging and grading, and anatomic location. However, for most of the advanced HNSCC, clinical staging does not correlate with treatment responses or prognosis and pre-operative clinical assessment of tumour and nodal involvement is often in disagreement with pathological T and N stage^2^. Variability in prognosis and molecular profiles of different head and neck tumours have attracted much attention in recent years as tumour heterogeneity represents a grave challenge for oncologists and seems to be a critical component of therapeutic response, cancer recurrence and patient survival. HPV infection probably covers most of the HNSCC heterogeneity. Beyond the role of HPV, current molecular classification categorises HNSCC into classical (CL), basal (BA) and mesenchymal (MS) subtype. HPV + tumours are not gathered into one group but fall into the MS and CL subgroup^3^. Genomic and proteomic data obtained from many HNSCC patients have demonstrated that HPV‐positive (HPV + ) and HPV‐negative (HPV−) HNSCC are different clinical entities^4^; (Box 1). Although the role of HPV in dysregulation of DNA damage response (DDR) is well established^5^, the influence of HPV on triggering of different kinds of cell death in HNSCC is discussed in a lesser extent, which is unfortunate because many viruses, including HPV, have developed numerous strategies to modulate host cell death to persist in the host for a long time without being eliminated. Accordingly, a growing spectrum of evidence suggests that the HPV-derived oncoproteins, such as E6 and E7 or E5, can inhibit death receptor signalling^6^. Nevertheless, the effect of HPV infection on other cell death types is not often discussed.

Deep understanding of the sensitivity or resistance to a specific cell death type given by certain genetic background and/or microenvironment that occur during the HNSCC pathogenesis may reveal targets for novel therapeutic approaches. We suggest that different approaches should be employed for therapy of different subgroups of HNSCC patients as the predominant type of cell death can have multiple impacts on the therapeutic response as cell death acts as a second messenger that guides both immune system and tissue microenvironment to ensure tissue repair and homoeostasis^7^. In this review, we discuss the different types of cell death, their connection with HNSCC pathogenesis and possible therapeutic windows that result from specific cell death sensitivity in some subgroups of HNSCC.

Box 1 HPV: the resource of heterogeneity in HNSCC HPV positivity is detected in about 25% of HNSCCs^207^. HPV‐positive (HPV + ) and HPV‐negative (HPV-) HNSCCs are derived from different anatomical locations (HPV‐negative cases are particularly located within the oral cavity, hypopharynx and larynx) and also have different mutation profiles, molecular characteristics, immune landscapes and clinical prognosis^208,209^. The immunologic profile of HPV + HNSCC was associated with a better outcome^24^. Accordingly, many studies demonstrated better response to therapy in HPV + HNSCC patients^4^. HPV infection also influences the genetic landscape of HNSCCs tumours and their predisposition to cell death. Despite the p53 tumour suppressor is the best-known target of HPV protein E6, some active p53 may still occur in HPV + HNSCC because these tumours usually harbour the wild-type form of the *TP53* gene*.* On the other hand, HPV-unrelated HNSCCs often have p53 mutations^14,15^. In HPV-negative HNSCC, deletion of 9p21–22 occurs early in cancerogenesis and the function of the tumour suppressor p16 is lost. On the contrary, when HPV protein E7 inactivates the Rb protein, p16 is overexpressed^210^. Proteins p16 and p53 are deeply involved in senescence and apoptosis.

## Intrinsic apoptosis

Apoptosis is a form of regulated cell death demarcated by the mitochondrial outer membrane permeabilization (MOMP) and accelerated by executioner caspases, mainly caspase-3. Once activated, caspase-3 cleaves target substrates such as poly(ADP-ribose) polymerase (PARP) and Lamin B which leads to the demise of the cell. While MOMP is essential for intrinsic cell death, the same is not true for caspases^8^. Nevertheless, the activity or inactivity of caspases provides a mechanism, which determines whether mitochondria initiate an immunologically silent or a pro-inflammatory type of cell death. If caspase activity is blocked following MOMP, cell death is accompanied by a type I interferon (IFN) response that alerts the immune system^9,10^. This response can be managed by mitochondria-dependent activation of the cGAS/STING pathway. Engagement of caspase-independent cell death displays potent anti-tumourigenic effects, often leading to complete tumour regression in the organism with intact immunity^9^. However, HPV + HNSCC cells respond poorly to activators of the cGAS-STING pathway. The attenuation of IFN responses results from the direct blockade of STING by viral protein E7^11^. Despite this fact, HPV + cancer cells can be still more easily recognisable by the immune system as HPV antigens could be presented together with common danger signals from dying cancer cells^12^.

HPV replication occurs in terminally differentiating epithelium and requires the activation of cellular DNA replication proteins. However, unplanned DNA replication can result in apoptosis and therefore the viral E6 protein induces the degradation of tumour suppressor p53 and the pore-forming protein BAK to prevent apoptosis^13^. Nevertheless, active p53 may still occur in HPV + HNSCC because these tumours usually harbour the wild-type form of the *TP53* gene. On the other hand, in HPV- HNSCC, p53 is mostly mutated^14,15^. Despite the wild-type form of the *TP53* gene, persistent infection with high-risk HPV presents a major risk factor in HPV-associated cancers. It was shown that NF-κB activity is involved in the establishment of persistent HPV infection as activation of NF-κB by HPV proteins limits viral replication through degradation of protein E1^16^. Unfortunately, NF-κB signalling can contribute to cisplatin resistance and cancer cells survival. Chemoresistant HNSCC cells with active NF-κB signalling respond to chemotherapy by promoting histone deacetylation and generation of heterochromatin. Therefore, targeted inhibition of histone deacetylases may be used as a possible therapeutic strategy for disrupting tumour resistance caused by NFκB^17^. NF-κB-mediated stabilisation of SNAI2 (Slug) can also underlie the inflammation-induced epithelial–mesenchymal transition (EMT) and metastasis in HNSCC^18^. On the other hand, NF-κB activation leads to reduced levels of nuclear BRCA1, impaired and prolonged DNA damage repair, prolonged accumulation of γH2AX foci and increased genomic instability^17^. As NF-κB induces the expression of various pro-inflammatory genes and participates in inflammasome activation^19,20^ and activation of the DDR has been linked to the increased presentation of major histocompatibility complex I (MHC I) molecules on the cell surface, this can result in increased recognition of cancer cells by cytotoxic T-lymphocytes^21^. Accordingly, HPV-positivity was correlated with increased T-cell infiltration, increased immune cytolytic activity, T-cell-inflamed immune microenvironment, the higher diversity of T-cell receptors, immune effector cell activation and improved response to anti-PD-1 therapy^22^. Infiltration of tumours with CD8 + cytotoxic T-lymphocytes has been associated with a favourable prognosis in several tumour types and may represent a predictive biomarker for cancer immunotherapy^23^. Consequently, the immunologic profile of HPV-positive HNSCC was associated with a significantly better outcome^24^. The HPV-specific immune response is also suggested to play a role in the significantly better response of HPV-positive patients to radiotherapy^25^. This higher sensitivity does not probably result from increased apoptosis or permanent G1-arrest but is rather associated with high levels of residual double-strand breaks and extensive G2-arrest^26^ and can be connected with a radiation-induced loss of cell surface CD47 enhancing the immune-mediated clearance of HPV + cancer cells^27^. Further evidence of the easier immune recognition is the fact that DNA damage generally induces higher expression of multiple ligands activating receptors of NK cells, such as NKG2D or DNAM1^21,28,29^. The DDR-mediated activation of NK cells may importantly contribute to the recognition and removal of pre-malignant and malignant cells^30^.

E1∧E4 is a viral protein profusely expressed in HPV-infected epithelia. It binds to the cytokeratin networks and in some cases induces their collapse. When cytokeratin is not present in the cell, E1∧E4 associates with mitochondria soon after its synthesis and induces the detachment of mitochondria from microtubules. This is followed by a severe reduction in the mitochondrial membrane potential and an induction of MOMP^31^. Interestingly, multiple studies have shown that HPV-positive oropharyngeal carcinomas are more likely to present reduced keratinization^32^ and more favourable outcome^33^. This can be connected to fast induction of MOMP in cells with low keratinization^31^. MOMP sensitivity can be especially supposed in HPV + mesenchymal subtypes (MS) of HNSCC as MS-signature contains downregulation of markers for epithelial differentiation and keratinization^3^. On the other hand, BA HNSCC subgroup exhibits high levels of epithelial keratinization and differentiation^34,35^. It is also possible that high cellular keratin content can confer some protection against MOMP triggered by mitochondria-binding chemotherapeutics as it was shown that high levels of keratin 6 cause chemoresistance to platinum drugs^36^.

Furthermore, it has been demonstrated that HPV-positive HNSCC cells use mitochondrial respiration and produce high levels of cytochrome c oxidase (COX), the key enzyme in the mitochondrial respiratory pathway. E6 oncoproteins also increased mitochondrial mass, protein levels of mitochondrial complexes (I to IV), ATP synthase and the voltage-dependent anion channel (VDAC). On the other hand, in HPV-negative HNSCC the mitochondrial OXPHOS is decreased and glycolysis is preferred due to non-functional p53^37–40^. Consequently, HPV-positive HNSCC may be more sensitive to mitochondria-targeted treatments, such as mitocans. Accordingly, HPV oncoprotein E7 enhances ceramide-mediated mitochondrial fission and lethal mitophagy in response to chemotherapy-induced mitochondria damage^41^.

The higher expression of VDAC, high mitochondria mass, and sensitivity to MOMP can also facilitate the therapeutic effect of cisplatin in HNSCC as cisplatin preferentially binds mitochondrial DNA and VDAC in the mitochondrial membrane^42^. TP53 mutations in HPV-negative HNSCC cells correlates with a metabolic shift toward glycolysis suggesting some beneficial effects of glycolytic inhibition during anticancer treatment. In contrast, wtTP53 expressing HPV-positive cells probably require inhibition of both, the mitochondrial respiration and glycolysis, to become sensitised to treatment^40^.

## Extrinsic apoptosis

Typically, extrinsic apoptosis is induced by the binding of the death ligands to death receptors, which then recruit adaptor molecules and initiator caspases to form the death-inducing signalling complex (DISC). Initiator caspases are then activated by proximity-induced cleavage at the DISC and in turn activate executioner caspases, which leads to the demise of the cell. In cancer cells, DISC formation is often weak and amplification of the death signal via the mitochondrial pathway is necessary for apoptosis.

From the view of death ligands and receptors, *TNFSF10* (encoding TRAIL) is the only gene found to be significantly altered in HNSCC. The most common alterations in components of the DISC found in HNSCC are amplifications of *FADD* gene (25% of HNSCC) and mutations of gene *CASP8* encoding procaspase-8 (10% of HNSCC). Interestingly, except HNSCC, there are no other tumour types with >10% incidence of *CASP8* mutations. The alterations in *CASP8* and *FADD* genes are significantly mutually exclusive, suggesting that they can be synthetically lethal^43^ as FADD and caspase-8 are needed for TRAIL-induced activation of NF-κB^44^.

In HPV-positive HNSCC, HPV proteins modulate apoptosis to prevent cell demise at early stages of viral infection. HPV16 E5 protein inhibits TRAIL signalling by interfering with the formation of DISC and subsequent cleavage of procaspases-8 and -3, as well as PARP. E5 also decreases the cell surface expression of the FAS receptor^45^. Similarly, it has been reported that E6 can inhibit apoptosis induced by TNF, FAS and TRAIL through the accelerated degradation of pro-apoptotic proteins such as FADD and/or procaspase-8 or through interactions with proteins that form DISC. Although E6 suppresses activation of both caspase-3 and caspase-8, it does not affect apoptotic signalling through the mitochondrial pathway fundamentally^46–49^. HPV-16 E6 and E7 oncoproteins upregulate *BIRC3* (*C-IAP2*) expression conferring the resistance to apoptosis^50^. In primary human keratinocytes, the HPV16 E7 protein alone increased both spontaneous and TNF-α-induced apoptosis but co-expression of E7 and E6 has cancelled the E7-mediated apoptosis^51^. The HPV E2 protein represses the expression of the viral oncogenes and activates viral DNA replication. An intact E2 gene is common in HPV16 positive oropharyngeal carcinomas but rare in HPV18-associated carcinomas. Cells expressing HPV18 E2 had perinuclear clustering of the mitochondria, higher ROS production, and loss of the cristae structure^52^. The presence of an intact E2 gene is associated with higher HPV viral load and improved clinical outcome^53^. The E2 protein induces apoptosis in both normal and HPV-transformed cells through activation of caspase-8^54,55^.

The finding that HPV16 E6 protein stimulates the degradation of the c-Myc oncoprotein seems rather surprising^56^. Nevertheless, c-Myc promotes oncogene-induced senescence (OIS) which is a critical tumour-suppressor mechanism preventing the transformation of cells. c-Myc promotes OIS through the transcriptional activation of p14/Arf/INK4B followed by p53 activation^57^. p14/Arf/INK4B is encoded by *CDKN2A*. HPV-positive HNSCCs rarely have *TP53* and *CDKN2A* mutations^58^ and overexpression of c-Myc can activate OIS in these cells. HPV16 can remain infectious for 2 weeks on senescent cells but require cell cycle re-activation for successful HPV infection^59^. Consequently, downregulation of c-Myc may be beneficial for HPV infection because of reduction of the senescent phenotype. On the contrary, most of the smoking-related HNSCCs demonstrate TP53 and CDKN2A inactivation^34^ and therefore do not force HPV to c-Myc downregulation. These tumours can exploit the oncogenic force of c-Myc without OIS induction. Accordingly, it was demonstrated that HPV + oral SCC patients with a history of tobacco use have a significantly poorer prognosis even compared to HPV − patients^60^. Nevertheless, *CDKN2A* copy number loss predicted poor survival independently of other clinical and treatment factors^61^.

## Crosstalk between apoptosis and autophagy

Caspases play a critical role in the crosstalk between autophagy and apoptosis as many autophagy-related proteins are recognised and cleaved by caspases (for example ATG3, ATG5, ATG16L1 or Beclin-1). In most cases, ATG proteins are degraded by caspases and the autophagic response is shut off^62^. In some special cases, the pro-autophagic proteins can be cleaved by caspases and converted into pro-apoptotic ones. For example, caspase-mediated cleavage of Beclin-1 inactivates autophagy and enhances apoptosis by promoting the release of pro-apoptotic factors from mitochondria^63^ and enhancing caspase-9 activity^64^.

The inhibition of essential apoptotic proteins during cancerogenesis can switch a cellular stress response from the default apoptotic pathway to autophagy. Accordingly, the role of autophagy regarding cell death should be rather protective. Nevertheless, the autophagy machinery can also interact with apoptosis in another way. As mentioned above, caspase-8 is activated by DISC. Another way of caspase-8 activation is managed by autophagosomes. This is executed by binding to the autophagic cargo receptor p62^65^. In the case that procaspase-8 is not activated in DISC, for example under high levels of c-FLIP_L_^66^, which is a frequent event in HNSCC^67^, this mechanism can rescue caspase-8 activity and enables cell death. This mechanism involves autophagosomes but not necessarily autophagic flux and degradation of cargo in autolysosomes. Consequently, late-stage autophagy inhibitors such as chloroquine or hydroxychloroquine can preserve this pro-apoptotic effect but can weaken some protective effects of autophagy. Thus, autophagic proteins either directly or through their caspase-cleaved fragments can effectively influence apoptosis. In addition to the above mentioned, ATG12 interacts with Bcl-2 and Mcl-1 and promotes apoptosis by acting upstream of mitochondria^68^. ATG12 also forms a complex with ATG3 regulating mitochondrial integrity during mitophagy^69^. Furthermore, cells undergoing BAX/BAK-mediated apoptosis show signs of activation of unc-51 like autophagy activating kinase 1 (ULK1) and marks of autophagy. This autophagic flux is triggered early in the apoptotic signalling and therefore an activation of the apoptosome or caspases are not necessary. This BAX/BAK-mediated autophagy inhibits the secretion of the pro-inflammatory IFN-β produced in response to mitochondrial damage which may be important for keeping immunological silence during apoptosis^70^.

## Necroptosis

Necroptosis is an alternative mode of regulated cell death displaying features of apoptosis and necrosis. Necroptosis is characterised by the formation of a complex called the necrosome that consists of the proteins RIPK1, RIPK3 (receptor-interacting serine/threonine–protein kinases 1 and 3) and mixed lineage kinase domain-like pseudokinase (MLKL). MLKL translocating towards the plasma membrane results in the formation of pores, causing an inflammatory response. Loss of RIPK1 and RIPK3 function by promoter hypermethylation strongly correlated with metastatic disease and poor prognosis in HNSCC patients^71,72^.

In normal circumstances, caspase-8 blocks necroptosis by cleaving RIPK1 and CYLD^73^. Inactivation of caspase-8 activity was not shown in HPV + HNSCC^74^. On the contrary, the stimulation of caspase-8 activity and concomitant re-localisation to the nucleus due to the interaction between viral E6 protein and caspase-8 was shown in both high and low-risk HPV types. E6 appears able to stimulate caspase-8 activity without apoptosis triggering^75,76^. This nuclear accumulation of caspase-8 was reported also in HPV-positive tumour cell lines and cervical cancer^77^. Protein E6 most likely recruits caspase-8 to the nucleus to perform functions that are beneficial either for the viral life cycle or in the maintenance of cell proliferation in E6 transformed cells. For example, active caspase-8 suppresses necroptotic cell death mediated by RIPK3 and MLKL and cleavage of RIPK1 by caspase-8 is a mechanism for dismounting death-inducing complexes, which is essential for limiting cell death in response to tumour necrosis factor α (TNFα)^78^. Moreover, HPV oncoproteins downregulate the expression of IFITM1 and RIPK3 to escape from IFNγ- and TNFα-mediated antiproliferative effects and necroptosis^79^.

**Fig. 1:**
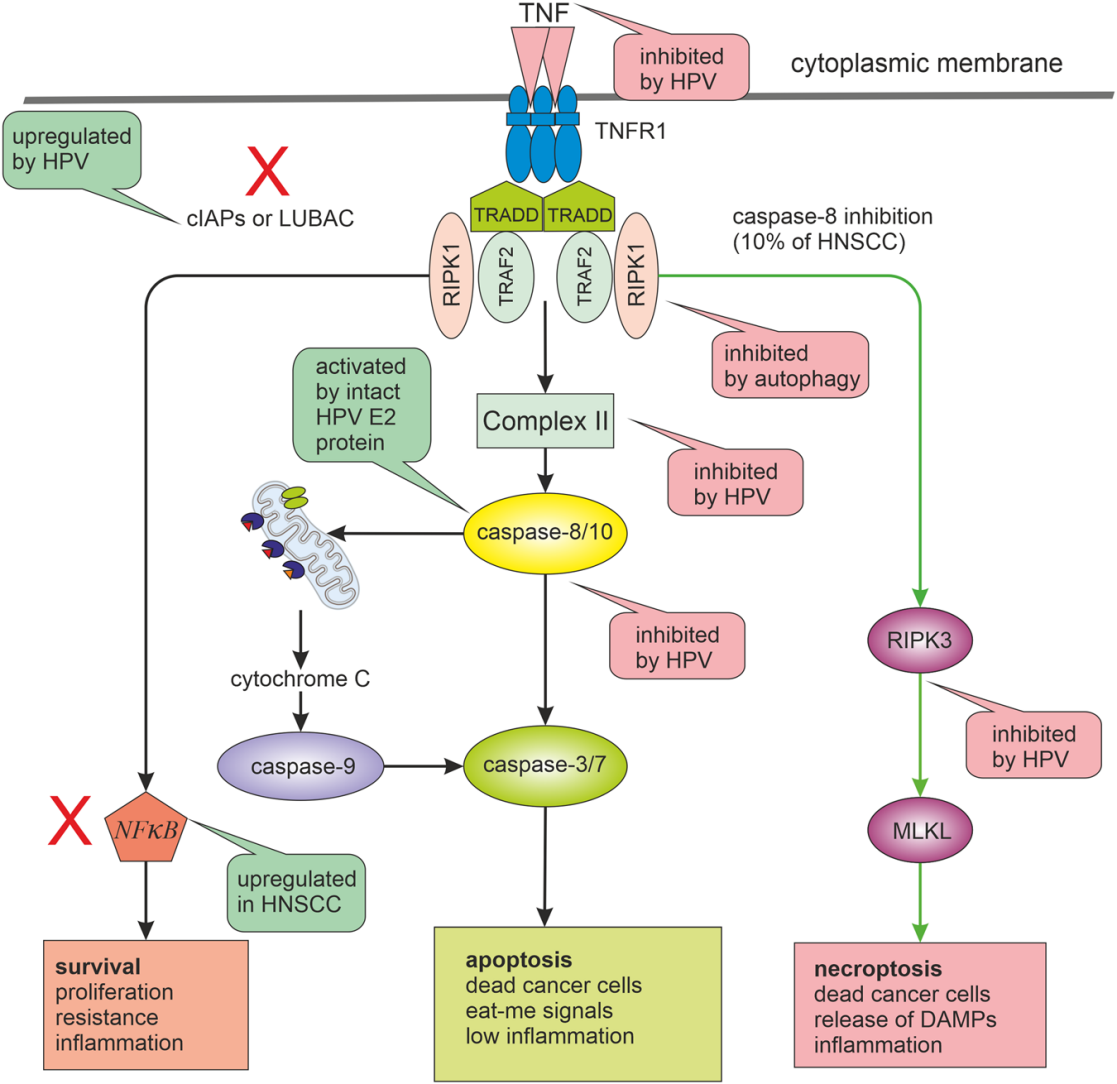
Alternative pathways of TNF signalling and their alterations in HNSCC. TNF signalling can lead to different results. It depends on the post-translational modification and activation of key molecules such as caspase-8, RIPK1 and NF-κB. While active caspase-8 triggers apoptosis and suppresses necroptosis, its inactivity (e.g. by mutations) leads to necroptosis through RIPK3 activation. If the RIPK1 signal is modified by the addition of ubiquitin, cell death can be attenuated, and the cell receives a signal for survival and proliferation by the transcription factor NF-κB. NF-κB pathway inhibitors such as LUBAC inhibitors or SMAC mimetics can sensitise HNSCC cells to necroptosis or apoptosis. For successful anticancer treatment response, the pathway marked by red exes should be inhibited (inhibition of autophagy should be also beneficial for necroptosis triggering). The TNF signalling pathways are also significantly influenced by HPV infection and by the genetic background of HNSCC. Green bubbles indicate activation and pink inhibition of the process. LUBAC linear ubiquitin chain assembly complex, c-IAPs inhibitors of apoptosis, RIPK receptor-interacting serine/threonine–protein kinases, MLKL mixed lineage kinase domain-like pseudokinase.

The gene *CASP8* encoding caspase-8 is mutated in 10% of HNSCC tumours analysed by The Cancer Genome Atlas^34^. Caspase-8 plays an important role in the apoptotic response of HNSCC to cisplatin and knockdown of caspase-8 substantially decreased apoptosis and cisplatin sensitivity^80^. But it is possible that HNSCC subtypes with caspase-8 mutations can be more sensitive to necroptosis triggering (e.g. by TNF-α) (see Fig. 1). Accordingly, a subgroup of oral cavity tumours with good clinical outcomes displayed inactivating mutations of caspase-8^34^. Consequently, targeting the necroptotic pathway seems to be a relevant therapeutic approach with compromised caspase-8 activity. However, the triggering of necroptosis should be done with caution. Necroptotic cells were shown to promote the migration and invasion of HNSCC cells in vitro through releasing DAMPs and RIPK1 can activate the NF-κB pathway in tumour cells which can lead to increased migration, invasion and proliferation^81,82^. Accordingly, TNF-α inhibits the growth of non-malignant cervical keratinocytes but stimulates proliferation of HPV-immortalised and cervical carcinoma-derived cell lines when mitogens such as epidermal growth factor (EGF) or serum are depleted^83^. Therefore, the triggering of necroptosis should be accompanied with suitable synergic treatment securing inactivation of the NF-κB pathway (see Fig. 1). It was shown that loss of caspase-8 function in combination with SMAC mimetic treatment sensitises HNSCC to radiation through induction of necroptosis, in case that RIPK3 function is maintained^84^. SMAC mimetics stimulate degradation of cIAP1 and cIAP2 and disrupt the activation of NF-κB^85,86^. Furthermore, the linear ubiquitin chain assembly complex (LUBAC) can also mediate NF-κB signalling and induce cell death resistance^87,88^. Inhibition of NF-κB activation by LUBAC inhibitors sensitised lung squamous cell carcinoma to cisplatin, suggesting a possible utilisation of these inhibitors and other NF-κB pathway inhibitors (e.g. curcumin) also in HNSCC patients^89–92^.

The important role in necroptosis induction may also play autophagy as autophagy inhibition or specifically ULK1 inhibition can enhance necroptosis by tumour necrosis factor (TNF) and toll-like receptor (TLR) ligands^93–95^. This pro-survival function of ULK1 is mediated via the phosphorylation of RIPK1 at Ser357^96^. The pro-survival function of autophagy is also supported by COP9 (Constitutive Photomorphogenesis 9) signalosome function. COP9 signalosome suppresses RIPK1-RIPK3–mediated necroptosis by regulating autophagosome maturation. Impaired autophagosome maturation causes necroptosis^97,98^. Proteins of COP9 signalosome are often overexpressed in cancer^99^. However, the crosstalk between autophagy and cell death can be more complex as the death-triggering function of ULK1 by the enhancement of PARP1 activity^100^, the scaffolding role of the autophagy proteins in balancing necroptosis and apoptosis (via the SQSTM1/p62-dependent recruitment of RIPK1)^101^, or the anti-autophagic function of RIPK1 (via the control of transcription factor EB^102^) were also described.

Necroptosis has dual effects as promoter or reducer of tumour growth in different types of cancer. As a backup form of cell death in cells with apoptosis failure, necroptosis can prevent tumorigenesis. Nevertheless, it can also trigger metastasis and immunosuppression. This can be caused by the release of IL-33 during necroptosis^103^. IL-33 seems to be involved in the shaping of the immunosuppressive environment during cancerogenesis^104,105^ and high expression of IL-33 in cancer-associated fibroblasts (CAFs) and tumour cells was associated with poor prognosis^106,107^. While these data suggest that IL-33 blockade may be beneficial for HNSCC patients, further investigations are needed to define all downstream signalling targets dependent on IL-33. Furthermore, the necrosome can promote oncogenesis via CXCL1 and Mincle-induced immune suppression^108^. On the other hand, necroptotic cells can provide both antigens and inflammatory cytokines to dendritic cells for antigen cross-priming which activates cytotoxic CD8 + T-lymphocytes demonstrating cytolytic effects and defence against tumorigenesis. RIPK1 expression and NF-κB activation are essential for this tumour suppressive mechanism^109^. Induction of necroptosis was also shown to induce anti-tumour immunity in an HMGB1-, nucleotide- and T-cell-dependent manner^110^.

## Pyroptosis

**Fig. 2:**
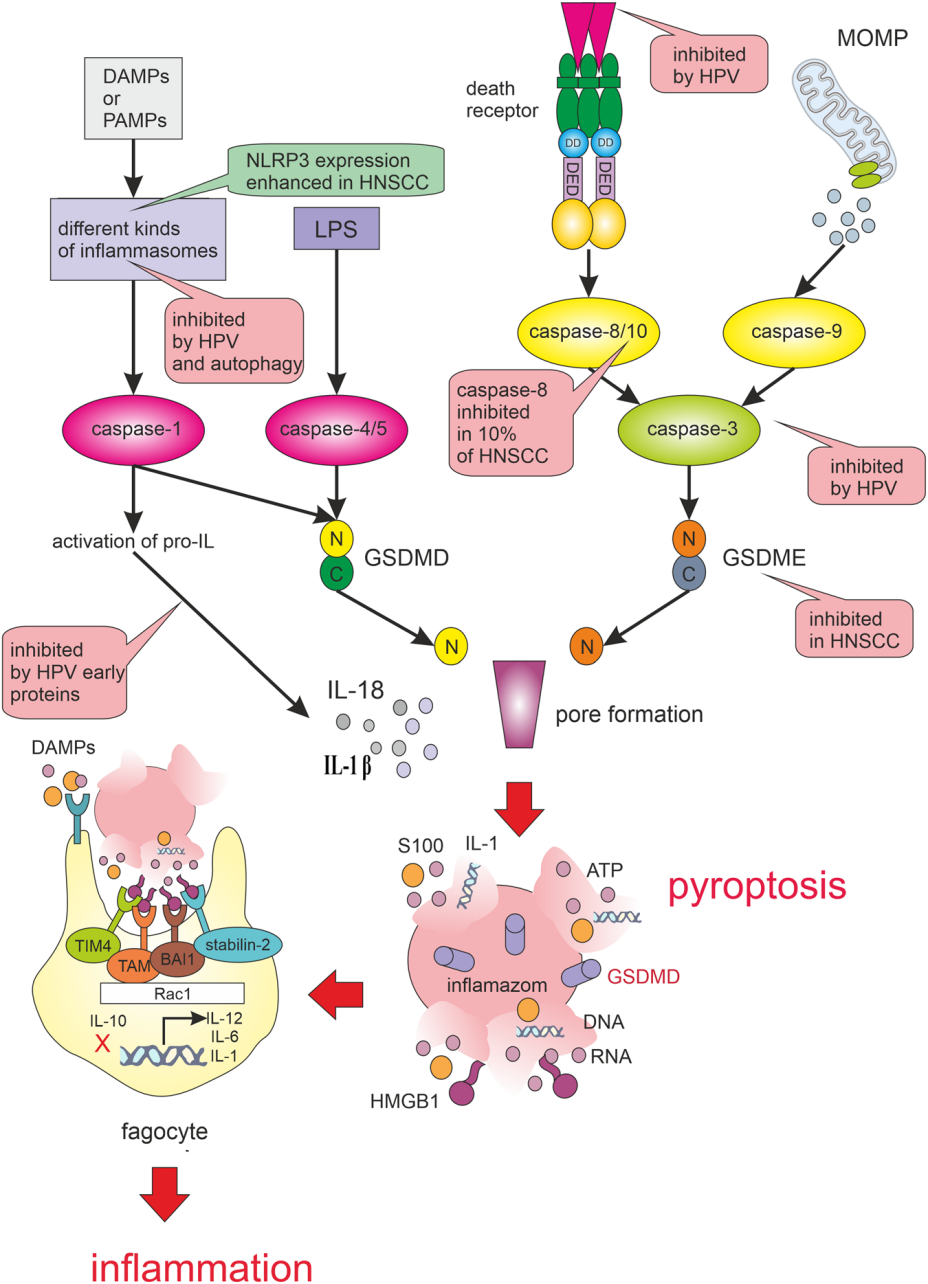
Pyroptosis pathways. Pyroptosis is triggered when damage-associated molecular patterns (DAMPs) or pathogen-associated molecular patterns (PAMPs) activate the inflammasomes. Active inflammasomes lead to the cleavage and activation of caspase-1. The activated caspase-1 cleaves Gasdermin D (GSDMD). The N-fragment of GSDMD form cell membrane pores resulting in pyroptosis. Activated caspase-1 also promotes the maturation and secretion of interleukins IL-1β and IL-18. When pathogen-derived lipopolysaccharide (LPS) binds to the precursor of caspase-4/5, it can also cause GSDMD cleavage and induction of pyroptosis. Another way to activate pyroptosis is caspase-3/Gasdermin E (GSDME) pathway. Caspase-3 can also be activated by mitochondrial outer membrane permeabilization (MOMP) and death receptor pathways. The activated caspase-3 then cleaves GSDME and produces GSDME N-fragments, The N-fragment of GSDME form cell membrane pores resulting in pyroptosis. Pyroptosis results in membrane rupture and the release of DAMPs that are detected by phagocyte receptors, such as TIM 4 (T cell immunoglobulin mucin receptor 4), BAI 1 (brain-specific angiogenesis inhibitor 1), stabilin-2 and TAM (Tryo3-Axl-Mer receptor). DAMPs recognition leads to the production of pro-inflammatory interleukins (e.g. IL-1, IL-6) and IL-12, which activates NK cells and induces the differentiation of naive CD4 T cells. The pyroptotic signalling pathways are significantly influenced by HPV infection and by the genetic background of HNSCC. Green bubbles indicate activation and pink inhibition of the process.

Pyroptosis is initiated by inflammatory caspases (1, 4 and 5) upon activation of the canonical or non-canonical inflammasome pathways. In the canonical inflammasome pathway, caspase-1 mediates the cleavage of gasdermin D (GSDMD) and the maturation of pro-inflammatory interleukins (IL-1β and IL-18). GSDMD pores then induce cell lysis, cell death and the leakage of intracellular components into the extracellular space. The non-canonical inflammasome pathway can be initiated by the direct binding of caspase-4 and -5 to lipopolysaccharide from Gram-negative bacteria^8^. Another way to activate pyroptosis is caspase-3/Gasdermin E (GSDME) pathway. Caspase-3 can be activated by mitochondrial intrinsic and death receptor pathway. The activated caspase-3 then cleaves GSDME. Cleaved GSDME N-fragments form pores in the plasma membrane, causing pyroptosis^111^ (see Fig. 2). This activation of GSDME may divert TNF-induced apoptosis to pyroptosis. Because GSDME expression is often silenced in tumour cells, which is not the case of healthy cells, this caspase-3 activity may be responsible for serious side effects of many chemotherapeutic regimens^112^. Pyroptosis can also be activated by caspase-8 and subsequent cleavage of GSDMD^113^. In the absence of GSDMD, caspase-1 can activate caspase-8, caspase-3, and caspase-7 and induce apoptosis, making apoptosis a backup programme for dysfunctional pyroptosis. Accordingly, tissue samples of HNSCC without the presence of lymph node metastasis showed high expression of caspase-1^114^. During apoptosis, caspase-3 and -7 specifically block cleavage of GSDMD^115^. In contrast, expression of inactive caspase-8 or pan-caspase inhibition induces the formation and subsequent activation of caspase-1. This mechanism triggers pyroptosis in cells with dysfunctional apoptosis^116^. Caspase-1 is also involved in the facilitation of cytoprotective autophagy during hypoxia-induced mitochondrial stress by activating LC3 and Beclin-1 and favouring clearance of damaged mitochondria^117^. In the feedback loop, autophagy downregulates inflammasomes as activation of autophagy by inflammatory signals limits IL-1β production by targeting inflammasomes for destruction. Both the AIM2 and NLRP3 inflammasomes can be recruited to p62 and engulfed by autophagosomes^118^. On the other hand, inhibition of autophagy enhances inflammasome activity^118^. Many studies have suggested that mitochondrial defects may promote inflammasome activation through excessive ROS production, mitochondrial cardiolipin exposure or release of mitochondrial DNA^119^.

Inflammation belongs among major causes of HNSCC cancerogenesis. Accordingly, inflammasome NLRP3 expression was enhanced in human HNSCC tissues and the IL-1β concentration was increased in the peripheral blood of these patients^120^. The increased expression of NLRP3 was also associated with tumour growth, invasiveness, metastasis, development of cancer stem cells (CSCs) and their self-renewal in HNSCC^121–123^. Furthermore, it has been reported that high NLRP3 expression is associated with poor clinical outcome in 5-FU-treated oral squamous cell carcinoma (OSCC) patients and NLRP3 knockdown increased 5-FU-induced apoptosis in OSCC cells^124^. Blockage of the NLRP3 inflammasome/IL-1β pathway by MCC950 improved anti-tumour immune responses in an HNSCC mouse model^120^ and blockade of the IL-1β pathway by biopharmaceutical drug anakinra has overcome erlotinib resistance in HNSCC xenografts^125^. On the other hand, alcohol has been shown to promote both the release of IL-1β and pyroptosis^126^. Infection of human keratinocytes with HPV16 also induced the secretion of IL-1β. Yet, upon expression of the viral early genes, IL-1β transcription is blocked, because HPV16 derived E6 protein can antagonise IL-1β production by inhibiting IRF6 transcription and upregulation of sirtuin 1 (SIRT1)^127,128^. Knockdown of SIRT1 upregulates AIM2 expression and triggers pyroptosis^128^. SIRT1 expression was associated with good prognosis in HNSCC patients^129^. Nevertheless, cigarette smoke impairs SIRT1 activity and promotes pro-inflammatory responses in epithelial cells^130^. HPV E7 may also inhibit pyroptosis by promoting TRIM21-mediated degradation and ubiquitination of the IFI16 inflammasome^131^.

Although pyroptosis seems to have rather tumour-promoting effects in HNSCC, the exogenous activation of pyroptosis has recently been shown to trigger powerful anti-tumour effect^112^. As many tumour cells have an innate resistance to apoptosis, the induction of pyroptosis may provide an efficient cancer therapy strategy. Indeed, interventions with some chemotherapeutic agents cause a switch from caspase 3-dependent apoptosis to pyroptosis by activation of GSDME^112,132^ and unleashing of inflammasome activation augments the efficacy of some immune checkpoint inhibitors^133^. GSDME also enhances the number and activity of tumour-infiltrating natural-killer (NK) and CD8 + T-lymphocytes, as well as phagocytosis of tumour cells by tumour-associated macrophages. Moreover, granzyme B produced by NK cells also activates pyroptosis in target cells by cleaving GSDME at the same site as caspase-3, thereby establishing a positive feedback loop. However, this mechanism of tumour suppression is abrogated in perforin-deficient mice or mice without killer lymphocytes^134^. Caspase-3 activation and cytochrome c release in response to apoptotic stimuli are significantly reduced in GSDME-deficient cells^135^. Uncleavable or pore-defective GSDME proteins are also not tumour suppressive and many cancer-associated GSDME mutations reduce GSDME function, suggesting that GSDME inactivation is a strategy developed by cancer cells to reach the immune evasion^134^. A decrease in GSDME expression and function was shown in radioresistant HNSCC^136^.

## Ferroptosis

**Fig. 3:**
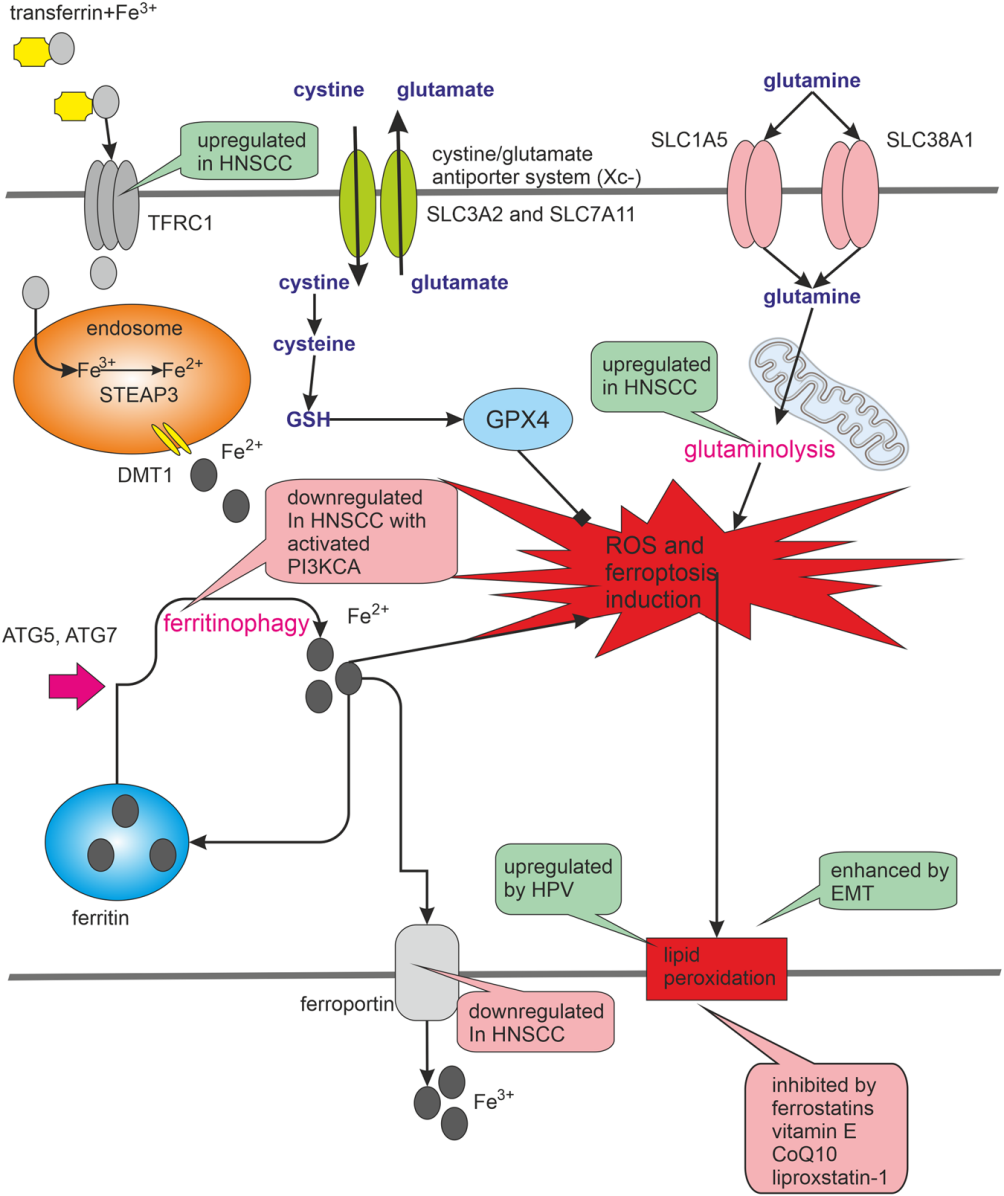
Ferroptosis. Ferroptosis is characterised by iron-induced lipid peroxidation. The intracellular concentration of iron can be affected by the activity of transferrin, transferrin receptor and ferroportin, or by the release of iron from ferritin, which is often managed by ferritinophagy. HNSCC cells often manifest an increased intracellular iron concentration due to a high level of TFRC1 (transferrin receptor 1 responsible for cellular iron uptake) and a low abundance of ferroportin (responsible for iron efflux). The non-physiological degree of lipid peroxidation and the initiation of ferroptosis is prevented by glutathione and glutathione peroxidase 4 (GPX4) activity. Ferroptosis could be also prevented with chelating agents or vitamin E, which casts a bad light on the benefits of antioxidants in the treatment of some ferroptosis-prone cancers. Glutamine and glutaminolysis also play a crucial role in the activation of ferroptosis. PI3KCA is among the most frequently mutated and activated genes in HNSCC. PIK3CA activation can lead to increased mTOR activity and decreased autophagy. Consequently, the presence of PI3KCA activation may predispose these cancer cells to avoid autophagy, ferritinophagy and ferroptosis. The ferroptotic signalling pathways are significantly influenced by HPV infection and by the genetic background of HNSCC. Green bubbles indicate activation and pink inhibition of the process.

Ferroptosis is independent of caspase activity. Instead, ferroptotic cells die following iron-dependent membrane lipid peroxidation. Importantly, tumour cells capable of evading other types of cell death probably maintain or acquire a sensitivity to ferroptosis. Given that HNSCC cells often manifest an increased intracellular iron concentration due to a high level of TFRC1 (transferrin receptor 1 responsible for cellular iron uptake)^137^ and a low abundance of ferroportin (responsible for iron efflux)^138^, ferroptosis-inducing therapy can be expected to effectively induce cell death in HNSCC cells without affecting normal tissue; see Fig. 3. Interestingly, the *TFRC* gene is located in the genomic region frequently amplified in HNSCC (3q29)^139^.

The induction of ferroptosis can be reversed by glutathione peroxidase 4 (GPX4) and ferroptosis suppressor protein 1 (FSP1). The blockade of cystine-glutamate antiporter (Xc–) system and cystine import is important for the induction of ferroptosis as inhibitors of (Xc–) system including sulfasalazine and erastin have been shown to induce ferroptosis^140^. The system (Xc–) consists of two subunits, SLC3A2 and SLC7A11. The expression of the SLC7A11 subunit is suppressed by protein p53 that inhibits cystine uptake and sensitises cells to ferroptosis. Even some mutated forms of p53, which are no longer able to induce apoptosis or senescence, do not lose their effect on the induction of ferroptosis^141^.

Tumour cells having mesenchymal features (tumour cell lines of mesenchymal origin, epithelial tumour cell lines that have undergone EMT and tumour cells exhibiting mesenchymal state-mediated resistance to anti-tumour therapy) appear to be particularly sensitive to ferroptosis. These cells usually have a higher activity of enzymes that promote the synthesis and storage of long-chain polyunsaturated fatty acids (PUFAs), which are a source of lipid peroxidation during oxidative stress. As a result, such cells are highly sensitive to GPX4 inhibition and ferroptosis. The interrelationship of mesenchymal phenotype and sensitivity to lipid peroxidation appears to be a result of the high expression of the protein ZEB1 (Zinc finger E-box-binding homeobox 1), which functions in both EMT and lipogenic processes. Deletion of ZEB1 was able to remove sensitivity to GPX4 inhibition in these cells^142^. ZEB1 was identified as a key player in the inflammation-induced promotion of EMT in HNSCC^143^. Accordingly, the MS subgroup of HNSCC characterised as having an elevated expression of EMT-associated genes could be the most sensitive to ferroptosis. In epithelial cells, interactions mediated by E-cadherin suppress ferroptosis by activating the intracellular NF2 and Hippo signalling pathway^144^. Furthermore, HPV16-derived E6 and E7 oncoproteins induce expression of the EMT-activating transcriptional factors Slug, Twist, ZEB1 and ZEB2^145^.

Another mechanism underlying ferroptosis was observed after erastin treatment. Erastin can bind with VDAC2 on the mitochondrial outer membrane, where it alters membrane permeability and the ion selectivity of the channels (allows only cations to move into mitochondria), causing mitochondrial dysfunction and ROS release that ultimately leads to glutathione (GSH) depletion and ferroptosis^146^. Recently, it was also found that activation of ferroptosis by erastin increases the level of lysosomal-associated membrane protein 2a (LAMP2), thereby promoting chaperone-mediated autophagy, which in turn promotes the degradation of GPX4^147^. Ferroptosis seems to be also tightly associated with ferritinophagy as inhibition of ferritinophagy by blockage of autophagy or knockdown of NCOA4, which mediates the selective autophagic degradation of ferritin, abrogated the accumulation of ROS and cellular labile iron^148^. HPV16 oncoproteins mute the host autophagic response at different levels of the autophagic pathway. E5 interferes with the phagophore assembly, while E6 and E7 inhibit autophagosome/lysosome fusion^149,150^. This autophagy inhibition may provide some resistance against ferroptosis in HPV-positive HNSCC, but the exact influence of HPV on ferritinophagy is currently not clear because it was also shown that HPV16 E6/E7 oncoproteins can activate autophagy via accelerating autophagosome formation and degradation^151^.

*PI3KCA* is among the most frequently mutated and activated genes in HNSCC in both HPV-positive and negative diseases (56 and 34%, respectively)^34^. Interestingly, PIK3CA activation in HPV-positive HNSCC can lead to increased mTOR activity and decreased autophagy^152^. Consequently, the presence of PI3KCA activation may predispose these cancer cells to avoid autophagy, ferritinophagy and ferroptosis. It was shown that aspirin or its active metabolite salicylate induce autophagy by inhibiting the acetyltransferase activity of EP300^153,154^. This effect of aspirin may be especially beneficial for HNSCC patients with PI3KCA over-activation. Accordingly, the use of nonsteroidal anti-inflammatory drugs (NSAID), such as aspirin, caused improved survival among HNSCC patients with PIK3CA mutations. Among subjects with PIK3CA mutations or amplification, regular NSAID use (≥6 months) conferred markedly prolonged disease-specific survival and overall survival compared to non-regular NSAID users^155^. In our opinion, this could be partly a consequence of autophagy and ferroptosis reactivation. Autophagy induction by rapamycin also showed the synergistic effects with irradiation in oral squamous cell carcinoma cells^156^.

In the absence of glutamine or inhibition of glutaminolysis, blocked cystine import cannot induce ferroptosis. Since many types of tumours, including HNSCC, are dependent on glutaminolysis^157^ and glutaminolysis is necessary to induce ferroptosis, can these tumour cells be more sensitive to induction of ferroptosis? Glutamine is synthesized by the enzyme glutamine synthetase (GS) from glutamate and ammonia. Glutamate is generated from glutamine during glutaminolysis, which is a series of biochemical reactions degrading the amino acid glutamine to glutamate, aspartate, CO_2_, pyruvate, lactate, alanine and citrate^157^. Both glutaminase-1 (GLS1) and glutaminase-2 (GLS2) are involved in glutaminolysis, but only GLS2 mediates ferroptosis and is a transcriptional target of the p53 protein. Accordingly, GLS2 has been recognised as a tumour suppressor and GLS1 more as an oncoprotein^158^. GLS1 expression was correlated with a poor survival rate in HNSCC patients^159^. It was also demonstrated that high expression of system (Xc–) components and glutamine transporter ASCT2 is correlated with undifferentiated status in HNSCC^160^ and ASCT2-dependent glutamine uptake is involved in the progression of HNSCC^161^. Sulfasalazine is a specific inhibitor of (Xc–)-mediated cystine transport and inhibits the growth of HNSCC cells^162^. It was shown that the cytotoxicity of sulfasalazine relies on ASCT2‐dependent glutamine uptake and glutamate dehydrogenase (GLUD)‐mediated α‐ketoglutarate production. Therefore, the activity of glutaminolysis-related proteins, such as ASCT2 and GLUD, can be probably used as a biomarker to predict the efficacy of sulfasalazine therapy in HNSCC^160^. On the other hand, sulfasalazine resistant HNSCC cells were found to highly express aldehyde dehydrogenase ALDH3A1 which plays a key role in the protection of cells from lipid peroxidation. This resistance was reversed by knockdown of ALDH3A1 or by ALDH inhibitor dyclonine^163^. Several studies have also revealed that transporters ASCT2 and (Xc–) mediate resistance in HNSCC cells after cetuximab, cisplatin and AG1478 treatment^164–166^.

**Fig. 4:**
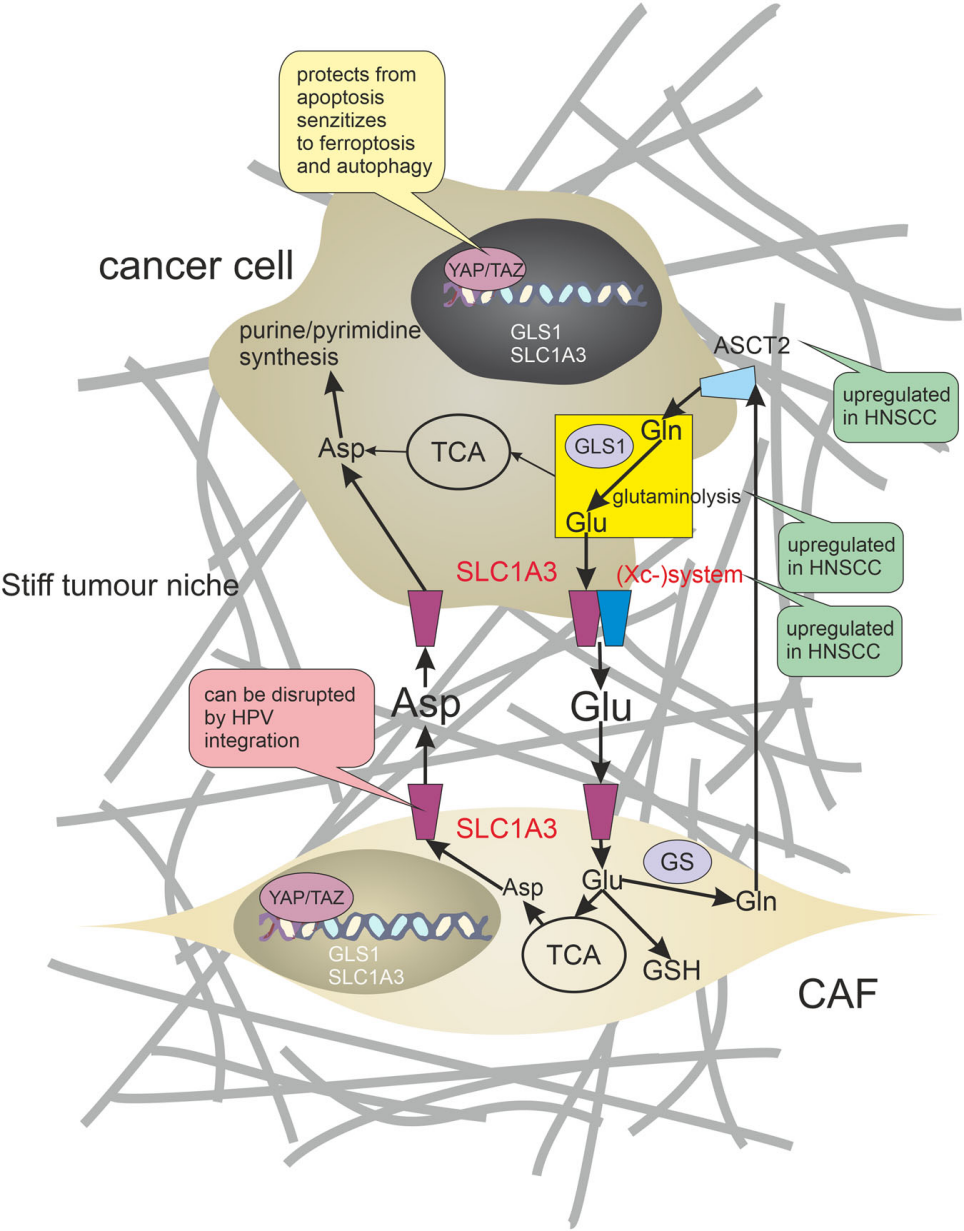
Metabolic symbiosis between cancer-associated fibroblasts and HNSCC cells. HNSCC cells undergo numerous metabolic changes including increased glutaminolysis. Glutaminolysis produces large pools of intracellular glutamate. Upregulation of the cystine/glutamate antiporter ((Xc–) system) and excitatory amino acid transporter (SLC1A3) promotes aberrant glutamate (Glu) release from cancer cells. Increased stiffness of extracellular matrix during tumour progression induces cancer-associated fibroblasts (CAFs) to import glutamate (through SLC1A3) and release aspartate (Asp) and glutamine (Gln) supporting cancer cell purine/pyrimidine synthesis. Glutamine synthetase (GS) is often overexpressed in these CAFs. CAFs can also promote chemoresistance through the production of glutathione (GSH). Establishing of this metabolic symbiosis is coordinated by a YAP/TAZ-dependent mechanotransduction pathway. Green bubbles indicate activation and pink inhibition of the process.

High concentrations of extracellular glutamate can block cystine uptake by the (Xc–) system and induce ferroptosis^167,168^. As cancer cells secrete high concentrations of glutamate through the activity of the cystine/glutamate antiporter (Xc–) system, they can be sensitive to ferroptosis when they have no support of other cells in the tumour microenvironment (TME). Nevertheless, increased stiffness of extracellular matrix during tumour progression induces CAFs to import glutamate (through SLC1A3) and release aspartate and glutamine supporting cancer cell proliferation; see Fig. 4. Glutamine synthetase (GS/GLUL) is often overexpressed in these CAFs^169^. In some cases, *SLC1A3* gene can be interrupted by HPV integration^170,171^. The metabolic symbiosis enables cancer cells to get rid of ferroptosis-promoting glutamate and to gain glutamine. This metabolic exchange can take place also between cancer cells and M2-macrophages^172^. Consequently, blocking the transport of glutamate into the CAFs or M2-macrophages may lead to enhanced sensitivity of glutamine-addicted cancer cells to ferroptosis. Nevertheless, this approach should be used with caution because increased levels of extracellular glutamate have been associated with the progression of cancer-induced pain^173^.

Establishing of glutamate metabolic symbiosis by metabolic reprogramming is coordinated by a YAP/TAZ-dependent mechanotransduction pathway^174^; see Fig. 4. Accordingly, levels of nuclear YAP/TAZ in fibroblasts associated with perineural invasion of HNSCC were higher than those in the stroma of normal mucosa^175^. YAP expression was elevated at the invasive front of HNSCC tumours^176^ and also as a consequence of PIK3CA overexpression^177^. Although YAP activation in tumour stroma can lead to avoiding ferroptosis caused by a high concentration of glutamate in TME, YAP itself can promote ferroptosis under some circumstances by upregulating ferroptosis modulators ACSL4 and transferrin receptor TFRC^144^. It seems possible that YAP/TAZ activity balances the tendency of cancer cells to undergo a distinct form of cell death as nuclear accumulation and activation of YAP/TAZ protect cells from apoptosis but sensitise cells to ferroptosis and autophagy^144,178^.

Some cells in TME, such as CD8 + T-cells, can induce ferroptosis in tumour cells and the insensitivity to PD-L1 inhibitors is often accompanied by resistance to ferroptosis^179^. One of the key factors causing the immunogenicity of ferroptotic cancer cells may be HMGB1^180^. Cancer cells undergoing ferroptosis release HMGB1 in an autophagy-mediated manner, when autophagy promotes HMGB1 acetylation, resulting in HMGB1 release^181^. Although HMGB1 serum and tissue levels were found to be elevated in HNSCC, they are associated rather with chemoattraction of regulatory T cells (Treg) and promoting of their immunosuppressive functions^182^. Ferroptosis was also associated with an increased expression of cyclooxygenase-2 and the release of prostaglandin E2 (PGE2) which facilitates immune evasion of tumour cells^183^.

## Targeted therapy of non-apoptotic programmed cell deaths in cancer

The development of new antineoplastic drugs targeting programmed cell death suitable for clinical use is a demanding and time-consuming process. Therefore, the effects of previously approved drugs on any type of programmed cell death should be intensively studied. The potential for such type of clinical use has been attributed to some cholesterol-lowering drugs, muscle relaxants, antimalarials or anti-rheumatic drugs such as sulfasalazine, lanperisone, statins, artesunate or aspirin.

Sulfasalazine (Azulfidine) has been repurposed to induce ferroptosis via inhibition of (Xc–) system. Cytotoxicity of sulfasalazine relies on glutamine uptake and α‐ketoglutarate production^162^. However, HNSCC cancer cells are capable of developing resistance. Resistance to ferroptosis induced by sulfasalazine may be overcome by ALDH inhibitor dyclonine or inhibition of CISD2^163,184^. Both pharmacological and genetic inhibition of SLC7A11 induce ferroptotic cell death and increase the cytotoxicity of cisplatin in HNSCC cells, which were resistant to cisplatin before this inhibition^185^. Activation of ferroptosis also appears to contribute to the efficacy of radiotherapy^186^ or some novel anticancer drugs with potential in HNSCC treatment, such as dihydroartemisinin^187^. (Xc–)-targeted therapy may also kill undifferentiated HNSCC cells expressing variant isoforms of CD44 (CD44v) and concurrently may sensitise the remaining HNSCC cells to available treatments including EGFR-targeted therapy^165^.

Lanperisone is a modified form of muscle relaxant tolperisone and selectively kills K-Ras-mutant cells through the induction of ROS, which is mediated through iron and ferroptosis pathways^188^. Ferroptosis may also be induced by statins as statins target the mevalonate pathway, which is crucial for GPX4 maturation^189^. This fact opens novel prospects of statins as therapeutic agents in cancer^190^. Another inductor of ferroptosis is probably artesunate and its derivatives. Artesunate is a medication used to treat malaria and can also produce ROS and cause oxidative stress in cancer cells. In pancreatic ductal adenocarcinoma, HNSCC and ovarian cancer cells, the anti-tumour effect of artesunate was mediated by induction of ferroptosis^185^.

Many proven anticancer drugs show a profound effect on programmed cell death. Dabrafenib, a B-RAF inhibitor approved for the treatment of melanoma, has also been shown to be a potent, high-affinity RIPK3 inhibitor that blocks TNF-α-induced necroptosis^191^. Other clinically approved drugs that may inhibit necroptosis include vemurafenib, sorafenib, pazopanib and ponatinib^192^. In contrast, induction of necroptosis has been identified as an important effector mechanism of oxaliplatin, mitoxantrone or 5-fluorouracil in anti-tumour activity^193,194^. In some tumour cells, inhibition of caspases is essential for the induction of necroptosis. Inhibitors of caspase activity can block caspase-8-mediated cleavage of RIPK1 and stabilise RIPK1-containing protein complexes. Inhibitors of caspase activity (e.g. IDN-7314, Z-VAD) are being tested as a novel approach in adjuvant chemotherapy of colorectal cancer showing resistance to 5-fluorouracil^194^. Induction of necroptosis also appears to be beneficial in the treatment of leukaemias and neuroblastomas^195,196^. Some results indicate that cisplatin and Val-boroPro (Talabostat) induce pyroptosis in cancer cells suggesting that they may provide additional advantages in the treatment of cancers with high levels of GSDME expression^197,198^. Ferroptosis can be induced by altretamine and sorafenib. Altretamine is an orally administered alkylating agent which is currently used as a secondary therapy for advanced ovarian carcinoma. It directly binds and inactivates GPX4^199^. Sorafenib is a kinase inhibitor approved for the treatment of renal cell carcinoma, hepatocellular carcinoma and thyroid carcinoma. Sorafenib induces ferroptosis independent from the oncogenic status of Ras, RAF, PIK3CA and p53 in cancer cell lines originating from different solid tumours^200^. Sorafenib increased the antiproliferative effect of cisplatin without affecting apoptosis in HNSCC cells, also enhanced HNSCC radiosensitivity^201^. Cisplatin alone was found to be a potent inducer of ferroptosis^202^.

The only clinically approved autophagy inhibitors nowadays are chloroquine (CQ) and hydroxychloroquine (HCQ)^203^. HCQ is currently in various stages of clinical trials as monotherapy or as part of combination therapy for solid tumours, however, pharmacodynamic studies suggest that the maximum permitted dose of HCQ (1200 mg/day) shows only slight inhibition of autophagy in vivo. This may be due to the reduced absorption of the drug into cells in an acidic environment (pH around 6.5), which is unfortunately typical of the tumour microenvironment^204^. Many CQ analogues with promising metabolic and antineoplastic effects are also in clinical trials^205^. Furthermore, some otherwise used compounds may have a profound effect on autophagy. For example, pro-apoptotic BH3-mimetic compounds such as ABT737, competitively disrupt the interaction between Beclin-1 and BCL2 or BCL-XL liberating Beclin-1 from an inhibitory complex and thus induce autophagy^206^. Aspirin induces autophagy via inhibition of the acetyltransferase EP300 and recapitulates features of caloric restriction^153,154^.

## Conclusion

Many HNSCC cancer therapies aim to induce apoptosis to suspend tumour growth. However, avoiding apoptosis is one of the key hallmarks of cancer and the presence of genetic heterogeneity and supporting TME severely limits the clinical efficacy of these approaches. Nevertheless, scientific consensus describes many different cell death subroutines with different regulatory pathways and cancer cells are probably not able to avoid all of cell death types at once. Therefore, a more focused and individualised therapeutic approach is needed to address the specific advantages and disadvantages of individual tumours. A full understanding of the sensitivity or resistance to specific cell death type given by certain genetic background and/or microenvironment that occurs during the HNSCC pathogenesis may reveal specific and effective targets for novel tailored therapeutic approaches. The genetic fingerprint of individual tumours can direct the development of novel agents to selectively hit the tumour cells while sparing the healthy ones. For example, HPV-positive HNSCC may be more sensitive to mitochondria-targeted treatments, such as mitocans, and the MS subgroup of HNSCC, having an elevated expression of EMT-associated genes, could be the most sensitive to ferroptosis. Consequently, the future development of agents that directly target cell death pathways could lead to disease regression even in patients with poor prognosis.
